# Investigation of an Optimal Material Addition Rate for Energy Consumption and Dimensional Accuracy in Fused Filament Fabrication of CFR-PEEK

**DOI:** 10.3390/polym16040492

**Published:** 2024-02-09

**Authors:** Kyudong Kim, Kijung Park, Hyun Woo Jeon

**Affiliations:** 1 Department of Industrial and Management Engineering, Incheon National University, 119 Academy-ro, Yeonsu-gu, Incheon 22012, Republic of Korea; sooe07@inu.ac.kr; 2 Department of Industrial & Management Systems Engineering, Kyung Hee University, Yongin-si 17104, Republic of Korea

**Keywords:** fused filament fabrication, CFR-PEEK, material addition rate, energy consumption, dimensional accuracy

## Abstract

The material addition rate (MAR) of fused filament fabrication (FFF) is an indicator of process efficiency varied by process parameter settings, which affects energy consumption and part quality in FFF. This study aims to identify the optimal MAR of FFF using carbon-fiber-reinforced polyether-ether-ketone (CFR-PEEK) by considering a trade-off between energy consumption and the dimensional accuracy of FFF outputs. A design of experiments considering two main process parameters is planned to print three sample types through FFF for CFR-PEEK. Then, the MAR (i.e., deposited material volume per build time) of FFF is obtained to derive individual regression models of energy consumption and the dimensional accuracy measured for each sample type. Furthermore, a trade-off between energy consumption and dimensional accuracy on the MAR is formulated to derive an optimal MAR for each sample type. The results show that FFF for CFR-PEEK has a trade-off between energy consumption and dimensional accuracy; there exists a specific MAR that maximizes the overall performance of energy consumption and dimensional accuracy for each sample type. The optimal MAR is the highest for the small volume sample, whereas it becomes the lowest for the vertical build orientation sample. This study suggests that the optimal MAR should be flexibly adjusted based on a fabricated part. The findings from this study also address the fact that decision-making for optimal FFF operations needs a transition from the identification of specific process parameter settings to the management of a proper process efficiency level in FFF.

## 1. Introduction

Additive manufacturing (AM) has received increasing attention as an innovative manufacturing technology in that AM provides new manufacturing opportunities with cost-effectiveness and environmental sustainability [1]. Among AM technologies, fused filament fabrication (FFF) is one of the most widely used techniques due to its cost-effective process, fabrication flexibility, and rapid processing for AM [2,3]. An FFF process extrudes a thermoplastic material through a heated nozzle to deposit the material layer by layer [4]. FFF is compatible with a wide range of polymer-based feedstock materials, from low-performance polymers such as polylactic acid (PLA) and acrylonitrile butadiene styrene (ABS) to high-performance polymers such as polyetherimide (PEI) and polyether- ether-ketone (PEEK) [5,6,7]. FFF has been employed for various applications, including biomedical [8], aerospace [9], and automotive [10] domains.

As the role of AM has been emphasized to realize sustainable manufacturing, recent studies have investigated energy performance in FFF to understand energy behavior and optimal AM operations for energy savings [1]. A concept of the process rate (i.e., added material volume or mass per printing time) of FFF has been employed in recent studies to effectively model energy performance in FFF [11,12,13,14]. This paper refers to the process rate of FFF as the material addition rate (MAR). Balogun et al. [15] proposed a general model of energy requirements in FFF, and they showed that the high cycle time and low MAR of FFF cause energy inefficiency. Liu et al. [12] addressed that the specific energy consumption of various AM devices decreases as the MAR increases. Lunetto et al. [13] derived regression models to predict the energy consumption of FFF using ABS and polycarbonate ABS on the MAR, and they claimed that the MAR is a comprehensive measure of complexity in FFF operations to effectively characterize the energy consumption of FFF. Hassan et al. [14] employed a regression model of electric power demand on the MAR of FFF for CFR-PEEK, and they simulated manufacturing scenarios with different FFF machine quantities to analyze a trade-off between energy costs and cycle time. Kim et al. [16] derived the regression models of power demand and energy consumption on the MAR of FFF for CFR-PEEK to simulate the energy performance of an AM system that handles multiple FFF machines and aircraft part designs.

Along with the energy consumption in FFF, the dimensional accuracy of FFF outputs has been considered one of the important aspects of evaluating part quality in AM. For this, existing studies have primarily investigated the effects of FFF process parameters on dimensional accuracy along with other performance measures [17,18]. Wang et al. [19] performed FFF experiments using ABS and identified that build orientation and layer thickness are significant factors for the dimensional accuracy of FFF outputs. Sood et al. [20] considered five FFF process parameters (i.e., layer thickness, part orientation, raster width, air gap, and raster angle) for ABS to identify optimal FFF parameter settings that maximize the overall dimensional accuracy of FFF outputs through the grey Taguchi method. Furthermore, they employed a machine learning approach using an artificial neural network technique to estimate the dimensional accuracy of FFF outputs. Sahu et al. [21] also considered five process parameters for ABS, which were considered in Sood et al. [20], to derive optimal parameter settings for the overall dimensional accuracy of FFF parts through the Taguchi method and the fuzzy logic method. Beniak et al. [22] performed an analysis of variance (ANOVA) to examine the effects of layer thickness and printing temperature on the dimensional accuracy of FFF using PLA and identified that thick layers and high printing temperatures worsen the dimensional accuracy of FFF outputs. Günay et al. [23] derived optimal layer thickness, printing speed, and orientation angle settings through ANOVA and grey relational analysis to maximize the overall dimensional accuracy of FFF outputs. They further identified the reproducibility of FFF through the process capability analysis of the printed samples. Park et al. [24] found that the layer thickness, build orientation, and printing speed of FFF for CFR-PEEK can differently affect the overall dimensional accuracy of FFF outputs depending on the part design.

The MAR of FFF indicates process efficiency in the AM process, which leads to less printing time for a given amount of material deposition at a higher MAR [13]. The FFF process with low process efficiency would negatively impact energy consumption in that the energy consumption of AM rapidly increases as the MAR decreases [25]. On the other hand, a high MAR that is critical for the energy efficiency of FFF may not be suitable for the part quality of FFF outputs. The fact that FFF does not have an adequate dimensional accuracy level for a fabricated part at a high printing speed [23] supports that a high MAR resulting from a high printing speed level can negatively affect the part quality of FFF outputs. Indeed, optimal FFF operations solely for energy consumption with compromised part accuracy are not desirable in practice, and both energy performance and part quality resulting from AM should be jointly considered to improve the overall performance [26].

The consideration of both energy consumption and part quality in FFF becomes more critical for a high-end polymer such as carbon-fiber-reinforced PEEK (CFR-PEEK). CFR-PEEK is one of the most popular high-performance polymers for medical and advanced engineering applications as a metal substitute due to its superior mechanical and chemical properties [27,28]. FFF using CFR-PEEK generally imposes high-level operational requirements (e.g., high processing time and expensive material costs) [24]. Given the operational expense of FFF for CFR-PEEK, a trade-off between energy consumption and part quality of FFF for CFR-PEEK becomes a critical issue; the energy consumption of FFF using CFR-PEEK is improved at a high MAR [16], whereas the dimensional accuracy of FFF outputs using CFR-PEEK deteriorates at a high printing speed [24] that leads to a high MAR. In this regard, understanding a proper process efficiency level for FFF using CFR-PEEK is necessary to have optimal AM operations that satisfy the overall best performance under a trade-off between energy requirements and part quality.

As a response, this study aims to identify an optimal MAR level for the FFF of CFR-PEEK to maximize the overall performance in both energy consumption and dimensional accuracy as a function of the MAR of the FFF. First, a full-factorial experimental design consisting of layer thickness at five levels and printing speed at six levels is planned to fabricate samples in three types, which are used to compare groups for part volume and build orientation. The energy consumption of each experimental sample is collected while the sample is being fabricated, and the overall dimensional error of each fabricated sample from the original dimensions is measured to represent the dimensional accuracy of FFF using CFR-PEEK. The MAR value of each fabricated sample is calculated using the consumed material volume and printing time of each fabrication to build the regression models of energy consumption and dimensional accuracy on the MAR. Then, a performance model that integrates the fitted regression models of energy consumption and dimensional accuracy is derived to identify an optimal MAR level of FFF for CFR-PEEK. The above procedure is performed for three sample types to identify changes in the optimal MAR level and to achieve the overall performance of both energy consumption and dimensional accuracy.

## 2. Materials and Methods

The following subsections illustrate the main steps to derive an optimal MAR that simultaneously considers both energy consumption and the dimensional accuracy of FFF for CFR-PEEK. Section 2.1 presents an experimental design to collect energy consumption and dimensional accuracy data for three sample types through FFF using CFR-PEEK. Section 2.2 performs regression modeling for energy consumption and dimensional accuracy on the MAR of FFF. Section 2.3 proposes a trade-off analysis procedure to derive an optimal MAR that simultaneously improves both performance measures.

### 2.1. Design of the Experiment

**Figure 1:**
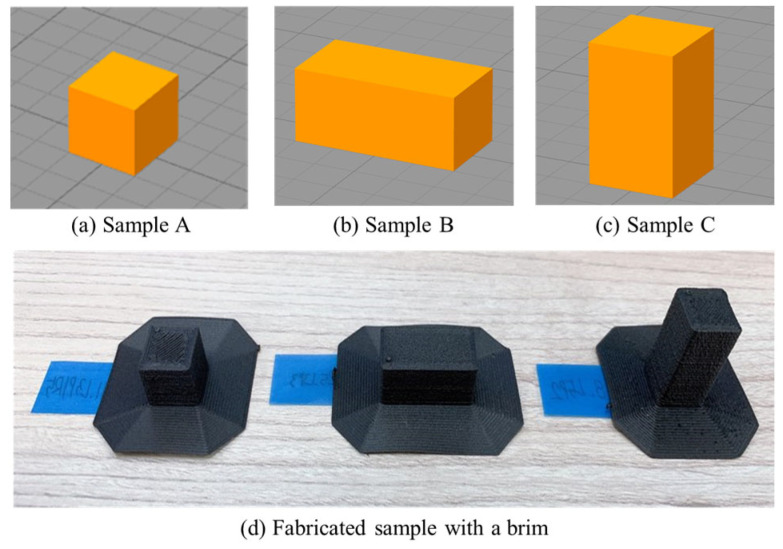
Three sample types for the experiments.

This study used three hexahedron design cases for experiments (see Figure 1). These sample types were employed as comparison groups for analysis. In Figure 1, Sample A illustrates a cube (i.e., 10 mm × 10 mm × 10 mm) to represent an object with a smaller volume and no build orientation effect (see Figure 1a). Sample B and Sample C represent a larger-volume object group (=2 cm^3^) than Sample A. In addition, they represent different build orientations of an object. Sample B and Sample C, respectively, represent horizontal and vertical orientation (i.e., 20 mm × 10 mm × 10 mm cuboid in Figure 1b and 10 mm × 10 mm × 20 mm cuboid in Figure 1c).

All experimental samples were fabricated by Apium P220 (Apium Additive Technologies GmbH, Karlsruhe, Germany) [29], which is an industrial FFF machine compatible with CFR-PEEK. TECAPEEK CF30 (Ensinger, Nufringen, Germany) [27], which has 1.38 g/${cm}^{3}$ in density, 6000 MPa in tensile modulus, and 112 MPa in tensile strength, was used as a material for the experiments. For each sample type, a full-factorial experimental design was planned to fabricate experimental samples by varying layer thickness and printing speed levels. These two FFF process parameters were chosen due to their significant effects on energy consumption and dimensional accuracy for CFR-PEEK outputs [14,16,24]. All possible combinations of layer thickness at five levels and printing speed at six levels were randomly organized for the experiments of each sample type (see Table 1). It is noted that 0.2 mm layer thickness and 1200 mm/min printing speed are the default process parameter settings for CFR-PEEK fabrication recommended by the manufacturer of the FFF machine. Experiments for Sample A in Figure 1 were considered a baseline experiment set to analyze a trade-off between energy consumption and dimensional accuracy based on the MAR of FFF for CFR-PEEK; a total of 90 samples (=five levels for layer thickness × six levels for printing speed × three replicates) were fabricated for all the process parameter combinations. In order to observe changes in the trade-off depending on volume variations and build orientation, 30 samples (=five levels for layer thickness × six levels for printing speed) for each type of Sample B and Sample C in Figure 1 were also fabricated, respectively. All process parameters except for the varied process parameters for the experiments were fixed according to the recommended parameter settings of the machine (see Table 1). In addition, a brim was set for each sample fabrication to ensure a complete AM process for each sample without detachment from the bed platform during the process (see Figure 1d). The process parameter settings of each experimental sample were controlled by the Simplify 3D slicing software [30].

**Figure 2:**
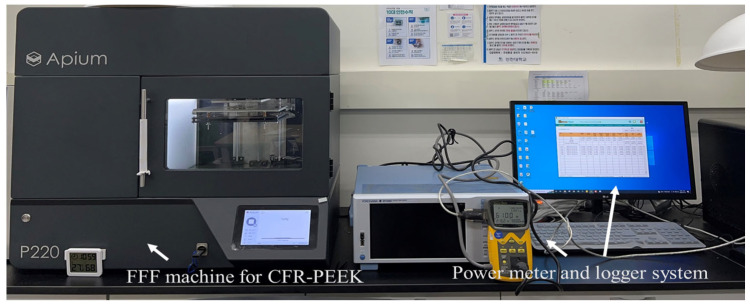
Equipment settings for measuring energy consumption in FFF experiments.

**Figure 3:**
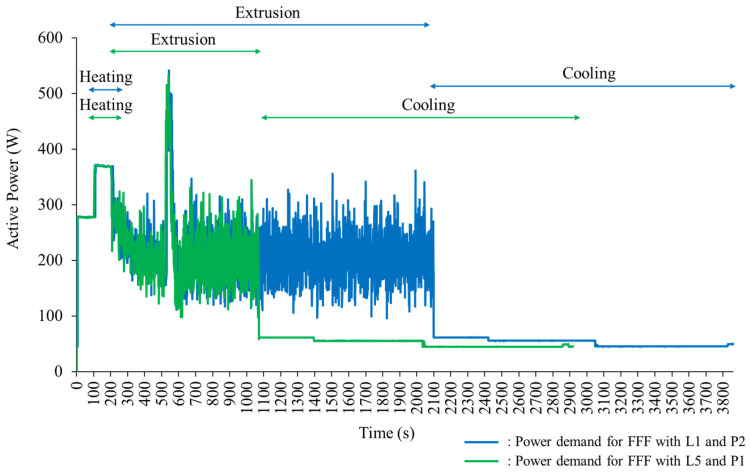
Examples of energy changes during FFF for CFR-PEEK.

Figure 2 illustrates the experimental environment of this study to collect energy data during each experiment. The energy consumption (W·h: watt-hour) of each experiment was recorded by the Wattman HPM-100A (ADpower, Pyeongtaek, Republic of Korea) [31] during the FFF process. The total energy consumption of each experimental sample during the material extrusion stage of the FFF process was considered for response data since power demands in the heating and cooling stages were not significantly varied across the experiments (see Figure 3).

The dimensions (i.e., width, length, and height) of the fabricated samples were measured by the Mitutoyo NTD13-P15M digital vernier caliper (Mitutoyo, Kawasaki city, Japan) [32]. The measured three axes of the fabricated samples were used to calculate the overall dimensional accuracy (error) using Equation (1). The root mean square error of each final output was calculated to represent the dimensional accuracy of the experimental sample based on dimensional differences between the original dimensions and the measured dimensions.

(1) $$Dimensional\;Accuracy\;(DA)=\sqrt{\sum_{i}{\left(M_{i}-A_{i}\right)}^{2}/3},$$

where $\;M_{i}$ is the measured value of dimension i (=x, y, and z-axis) for an experimental sample, and $A_{i}$ is the original size of dimension i for an experimental sample.

The total build time during the extrusion stage of each experiment and the total volume of the deposited material for each fabricated sample were also collected by referring to time in measured energy profiles and the Apium P220 data system, respectively. Table 2 summarizes the experimental data collected for this study.

### 2.2. Modeling Energy Consumption and Dimensional Accuracy on MAR

Individual regression models for energy consumption and dimensional accuracy on the MAR of FFF for CFR-PEEK were derived first based on the collected experimental data. The MAR of each experimental sample was calculated by dividing the total deposited volume by the build time (=v/t) to represent the amount of deposited material per time. Then, the energy consumption model for FFF in Equation (2) [16] was used to fit the energy consumption data of fabricated samples through linear regression.

(2) $$EC=\beta_{0(EC)}+\beta_{1(EC)}\cdot MAR_{inv},$$

where $MAR_{inv}=1/MAR$, $\beta_{0(EC)}$ is an intercept, and $\beta_{1(EC)}$ is a coefficient for the predictor.

The dimensional accuracy of FFF for CFR-PEEK was expressed as a function of MAR through a linear regression model in Equation (3). A linear regression model for dimensional accuracy was assumed by referring to the findings of Park et al. [24], which identified the statistical significance of layer thickness and printing speed on the dimensional accuracy of FFF using CFR-PEEK through ANOVA.

(3) $$DA=\beta_{0(DA)}+\beta_{1(DA)}\cdot MAR,$$

where $\beta_{0(DA)}$ is an intercept, and $\beta_{1(DA)}$ is a coefficient for the predictor.

The above regression models for energy consumption and dimensional accuracy were fitted using MINITAB 20.3 [33]. The statistical significance and prediction performance of the derived regression models were examined by *t*-test and *R*-squared, respectively. The modeling procedure in this section was applied to each sample type to derive fitted energy consumption and dimensional accuracy models.

### 2.3. Optimal MAR Based on the Trade-Off between Energy Consumption and Dimensional Accuracy

A trade-off analysis for each sample type was performed to characterize both energy consumption and dimensional accuracy on the MAR of FFF for CFR-PEEK. Since energy consumption and dimensional accuracy have the same objective direction (i.e., to be minimized) with different value scales, a normalization process in Equation (4) [34] was performed to make them have the same value scale for the experimental dataset of each sample type.

(4) $$y_{i}\prime =(max\left(y\right)-y_{i})/\left(max\left(y\right)-min(y\right)),$$

where $y_{i}\prime$ is a normalized response value, $y_{i}$ is an original response value for normalization, max⁡(y) is the maximum value of response variable *y*, and min⁡(y) is the minimum value of response variable *y*.

Then, the same regression procedure for the original data in Section 2.1 was performed to derive regression models for normalized energy consumption and dimensional accuracy. Then, regression models constructed for normalized energy consumption and dimensional accuracy were added to characterize the overall AM performance. Equation (5) shows a proposed AM performance model on the MAR of FFF.

(5) $$P=\beta_{0(EC\_Norm)}+\beta_{1(EC\_Norm)}\cdot 1/MAR+\beta_{0(DA\_Norm)}+\beta_{1(DA\_Norm)}\cdot MAR,$$

where *P* denotes the overall AM performance, $\beta_{0(EC\_Norm)}$ and $\beta_{0(DA\_Norm)}$ are constants for the regression models for normalized energy consumption and dimensional accuracy, and $\beta_{1(EC\_Norm)}$ and $\beta_{1(DA\_Norm)}$ are regression coefficients for normalized energy consumption and dimensional accuracy.

Through the normalization process, both energy consumption and dimensional accuracy are transformed from cost criteria (i.e., the smaller the better) to benefit criteria (i.e., the larger the better). It is assumed that energy consumption and dimensional accuracy are in a trade-off relationship depending on the MAR of FFF; as the MAR increases, the normalized energy consumption performance rapidly increases, whereas the normalized dimensional accuracy performance worsens. Then, the formula for *P* forms a concave function that reflects the trade-off, where *P* becomes maximum at a specific MAR. The optimal MAR value that maximizes the overall performance can be obtained by a differential equation of *P*. That is, the MAR value that satisfies dP/dMAR=0 becomes the optimal MAR considering the trade-off. Changes in the trade-off behavior and the optimal MAR of the sample types were analyzed to find implications regarding the role of the MAR for FFF operations.

## 3. Results

The regression models for energy consumption and the dimensional accuracy of each sample type are presented in Section 3.1. Section 3.2 shows the results of the trade-off analysis on the MAR.

### 3.1. Energy Consumption and Dimensional Accuracy Models for FFF Using CFR-PEEK

**Figure 4:**
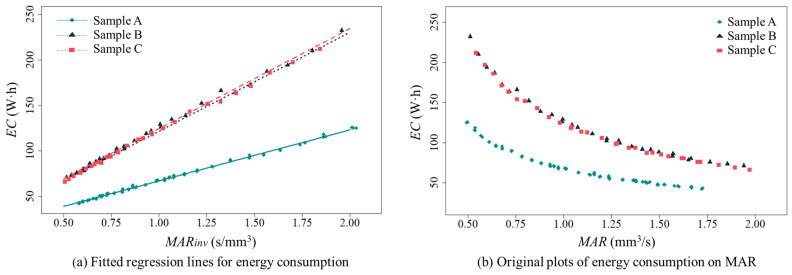
Energy consumption on MAR for each sample type.

Table 3 shows the statistical significance of regression models for energy consumption on the MAR of FFF for each sample type. Furthermore, Figure 4 illustrates regression lines of energy consumption on the MAR for each sample type. The regression results of energy consumption for all the sample types reveal that energy consumption is suitably fitted as a linear function of the inverse MAR; the energy consumption of FFF for CFR-PEEK nonlinearly decreases as the MAR increases (see Figure 4b). The inversed MAR is statistically significant in all the derived regression models, regardless of the sample types (see Table 3). Moreover, all the regression models have high explanatory power for a relationship between energy consumption and the inversed MAR.

In Figure 4a, Sample B and Sample C have almost the same energy consumption pattern, and their energy consumption is always higher than the energy consumption of Sample A for every inverse MAR. This result seems to be due to the part volume required for the FFF of Sample B and Sample C; Sample B and C have twice as large a part volume as Sample A. Accordingly, the regression coefficients for the Sample B and C cases are approximately twice as high as the regression coefficient of the energy consumption model for Sample A (see Table 3). This indicates that the energy consumption required for the FFF of Sample B and Sample C more rapidly decreases than for the FFF of Sample A as the MAR increases (see Figure 4b). The regression results indicate that energy consumption is significantly varied depending on the MAR of FFF and the fabricated part volume.

**Figure 5:**
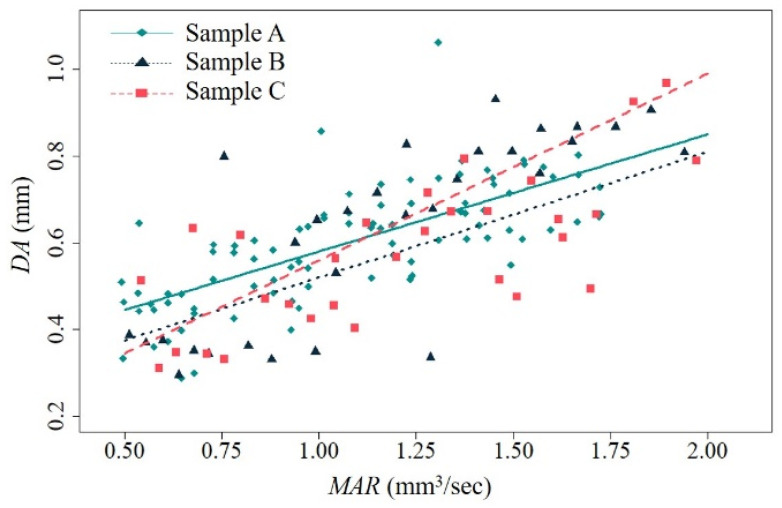
Dimensional accuracy on MAR for each sample type.

Table 4 presents the linear regression results of dimensional accuracy on the MAR of FFF for each sample type. Figure 5 shows the linear regression plots of dimensional accuracy on the MAR. The results show that the overall dimensional error of each fabricated sample statistically increases as the MAR of FFF increases. The *t*-test for $\beta_{1(DA)}$ in Table 4 shows that the MAR is a statistically significant linear predictor of the dimensional accuracy measure for each design case. However, the *R*-squared values of all the regression models are at a moderate level, possibly due to dimensional variations added by the manual measuring process using the vernier caliper and uncontrollable experimental factors in this study. Different from the regression results for energy consumption, the fitted regression lines of the Sample A and Sample B cases show a similar pattern. On the other hand, the regression coefficient for Sample C is the highest among the derived regression coefficients for the sample types; the negative effect of the MAR on the overall dimensional error of a fabricated part for Sample C is more critical than in the other cases as the MAR increases (see Figure 5).

Sample C, which is a variant of Sample A with an increase in the z-axis dimension from Sample A, has a low dimensional accuracy performance in FFF with process parameters that result in a high MAR. It is noted that the crossing point between the regression lines for Sample A and Sample C in Figure 5 is 1.13 mm^3^/s, approximately. The overall dimensional error of Sample C becomes greater than that of Sample A at the MAR, which is greater than 1.13 mm^3^/s. On the other hand, the dimensional error of Sample C is expected to be lower than that of Sample A when the MAR is less than 1.13 mm^3^/s. This indicates that a decrease in the MAR of FFF using CFR-PEEK can be effective in enhancing the dimensional accuracy of the fabricated part if the part height is increased from its original dimension. Alternatively, a change in the build orientation of Sample C can be an option to improve dimensional accuracy at the same MAR of FFF. Figure 5 shows that Sample B, which has the same part volume as Sample C, is expected to have better dimensional accuracy than Sample C at most MAR levels.

The regression models built for the energy consumption and dimensional accuracy of FFF for CFR-PEEK confirm a trade-off between those performance measures on the MAR. Figure 4b supports that energy consumption is in an inverse relationship with the MAR, whereas Figure 5 shows that dimensional error is in proportion to the MAR. The results show that there can be an optimal MAR at which both performance measures are suitably achieved by avoiding extreme losses in either energy consumption or dimensional accuracy performance.

### 3.2. Optimal MAR Considering Both Energy Consumption and Dimensional Accuracy

**Figure 6:**
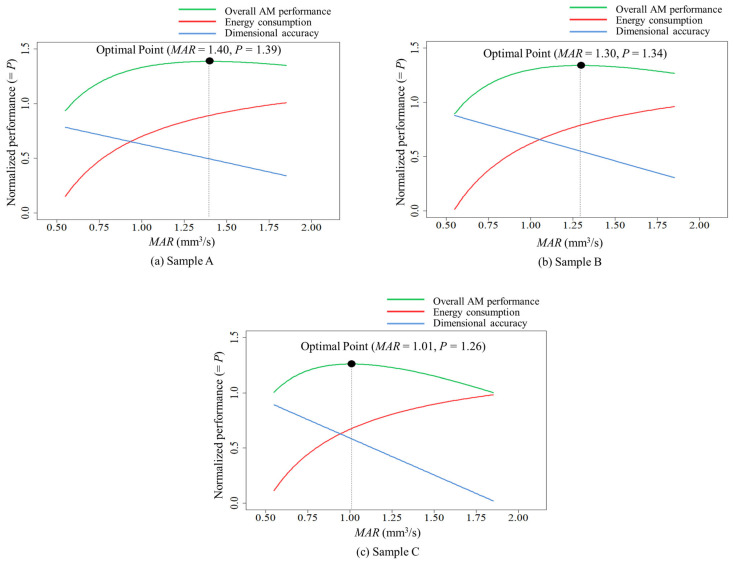
Optimal MAR to maximize overall AM performance.

Table 5 and Figure 6 show an optimal MAR for each sample type when the trade-off between the energy consumption and dimensional accuracy of FFF for CFR-PEEK is considered to maximize the overall performance. The normalized performance plots shown in Figure 6 clearly address the fact that the FFF of CFR-PEEK for all the sample types has a trade-off between energy and quality performance depending on the MAR.

The optimal MAR for the baseline case (i.e., Sample A) to maximize the overall AM performance of energy consumption and dimensional accuracy is 1.40 mm^3^/s, which is the highest process rate of all the sample types. This indicates that Sample A, requiring half the volume of the other sample types, can be optimally fabricated at a relatively higher process rate than the other sample types. In addition, the results in Table 5 and Figure 6 show that Sample B and Sample C require a different optimal MAR, although their designated part volumes are equal. The overall AM performance of Sample B is optimal at the MAR of 1.30 mm^3^/s, which is near the optimal MAR for Sample A. On the other hand, the lowest optimal MAR of all the types (=1.01 mm^3^/s) is required for Sample C.

The optimal MAR for Sample C is lower than that of Sample B, since the dimensional accuracy performance of Sample C decreases faster than that of Sample B (see Figure 6c) without significant variations in energy performance between Sample B and Sample C (see Figure 4b). The findings indicate that the build orientation of a fabricated part can be a critical factor in AM process efficiency. A change to horizontal build orientation to have a wide bottom area can be an effective strategy to pursue higher process efficiency in FFF if mechanical properties (e.g., tensile and compressive strength) affected by the build orientation are not critical for the fabricated part. On the other hand, a part design that has a height size relatively higher than sizes on the x-y plane may require the FFF with a relatively lower MAR to maintain the best energy and quality performance in the FFF if the FFF of the part requires its original build orientation due to mechanical functionality. The above results also suggest that the MAR of FFF for CFR-PEEK should be properly managed to improve the overall AM performance by jointly considering energy consumption and dimensional accuracy, depending on the part designs considered for AM.

Table 6 shows the process parameter combinations of the experiments that lead to the experimental MAR being close to the theoretical optimal value obtained for each sample type. For this, all the MAR values calculated from the experiments were rounded off to the first digit after the decimal point, and then the parameter combinations that result in the MAR value closest to the optimal MAR were identified for each sample type. For Sample A, the process parameter combinations specified in Table 6 are the experimental settings in which all the experimental replicates result in rounded-off MAR values equal to the optimal MAR. It is noted that the energy consumption and dimensional accuracy data specified for Sample A in Table 6 are averaged values for the associated replicates. The results imply that there can be multiple process parameter combinations to satisfy the overall best AM performance of energy consumption and dimensional accuracy. This indicates that the process parameters of FFF for the overall AM performance are not a fixed and single optimal process parameter set but a flexible and selective solution among the alternative parameter combinations leading to the optimal MAR.

## 4. Discussion

The resultant regression models of energy consumption show that the energy consumption of FFF for CFR-PEEK is expressed as a decreasing non-linear function of the MAR, as addressed in the existing studies. Indeed, process parameter combinations that lead to a short build time in this study show a high MAR and a resultant low energy consumption value in that the energy consumption of FFF for CFR-PEEK is significantly affected by printing time [14]. This energy behavior on the MAR is consistently observed in all the sample types, and the results support that an increase in AM process efficiency can greatly decrease the energy consumption of FFF for CFR-PEEK. In particular, Sample B and Sample C, which have the same part volume with different build orientations, show similar energy consumption plots on the MAR; their energy consumption is higher than the energy consumption of Sample A for every MAR. This indicates that the energy consumption of FFF for CFR-PEEK is not significantly varied by the build orientation but is affected by the designated part volume.

On the other hand, the dimensional accuracy of FFF for CFR-PEEK can be modeled as a linear function of the MAR. A high MAR is achieved when layer thickness and printing speed are set at high levels. However, these process parameter settings can negatively impact printing resolution and thereby may cause an increase in the overall dimensional error of a fabricated part. The negative impact of the MAR on dimensional accuracy is stronger for Sample C than for Samples A and B, which have a similar pattern of overall dimensional error on the MAR. A plausible reason for the worsened dimensional accuracy observed in Sample C compared to Sample B, which has the same design and volume as Sample C, is build orientation that increases the number of deposited layers. Sample C is considered a vertically oriented Sample B on the build platform. FFF has greater residual stress and heat shrinkage in the vertical direction (z-axis) than in the horizontal direction (x-y plane), and therefore this can cause more dimensional deviations in a part fabricated in a vertical orientation [35]. The results from the dimensional accuracy models indicate that build orientation is a more critical factor for the dimensional accuracy of FFF for CFR-PEEK than part volume.

The above energy consumption and dimensional accuracy characteristics of FFF for CFR-PEEK clearly show a trade-off on the MAR. The overall AM performance model, through the integration of the normalized energy consumption and dimensional accuracy models, presents that there is an optimal MAR at which FFF for CFR-PEEK is the most effectively operated given the trade-off of energy consumption and dimensional accuracy. Moreover, the fact that the optimal MAR varied by the sample type implies that the FFF for CFR-PEEK should be flexibly operated to achieve an optimal MAR targeted for part volume and orientation; relatively high AM efficiency can be pursued for a small volume part (e.g., Sample A) in the FFF for CFR-PEEK to have the best performance in energy consumption and dimensional accuracy. If the horizontal orientation of a part (e.g., Sample B) is allowed to increase the contract area on the build platform, FFF for CFR-PEEK can be managed to improve process efficiency that is expected to be lower in the original vertical orientation of the part (e.g., Sample C).

## 5. Conclusions

This study focused on a trade-off between energy consumption and dimensional accuracy of FFF outputs using CFR-PEEK to identify an optimal MAR to maximize the overall AM performance of both energy consumption and dimensional accuracy. For this, an experimental design considering five levels of layer thickness and six levels of printing speed for FFF using CFR-PEEK was planned to manufacture experimental samples of three types. Energy consumption and dimensional accuracy data measured for each experiment were collected as response data, and the MAR of each experiment was calculated to be used as input data. Herein, the MAR was employed to represent the process efficiency of FFF using CFR-PEEK, which is an operational consequence of selected process parameter settings, to effectively model energy consumption and dimensional accuracy for FFF.

Based on the collected data, individual regression models of energy consumption and dimensional accuracy on the MAR for the considered sample types were derived to confirm the impact of the MAR on the performance measures. Then, the original response data were normalized to derive individual regression models for normalized energy consumption and dimensional accuracy again, and they were integrated to represent the overall AM performance model of FFF for CFR-PEEK for the sample types, respectively. Finally, an optimal MAR to maximize the overall AM performance of each sample type given a trade-off between energy consumption and dimensional accuracy performances was identified to address the role of the MAR in successful FFF operation.

The findings of this study suggest that decision-making in FFF operations should be viewed as maintaining optimal process efficiency instead of selecting optimal parameter settings. Previous attempts to suggest optimal FFF operations in the literature focused on identifying specific process parameter settings that maximize a specific performance measure. This was reasonable when the AM decision-maker was only interested in optimizing a single performance measure. However, it would be difficult to determine specific process parameter settings if multiple performance measures with complex trade-offs should be jointly considered to improve the overall AM performance. In addition, optimal process parameter settings for a specific FFF machine cannot ensure the optimal performance of another FFF machine due to different machine specifications. Thus, FFF operations are required to be considered as a generalized problem of process efficiency rather than specific process parameter selection, and any process parameter combination associated with an optimal MAR can be a solution for the best AM operation based on an AM strategy. For example, if an AM decision-maker wants to significantly save energy consumption to fabricate parts by compromising dimensional accuracy to some degree, process parameter combinations generating a higher MAR in any FFF machine would be preferable.

This study contributes to decision-making for AM as a process efficiency-based AM approach to generally model a trade-off between energy consumption and dimensional accuracy. Nevertheless, this study should be further extended to understand the dynamics of process efficiency not only for various design cases but also for more conflicting AM performance measures in practice. In addition, operation strategies to flexibly handle the MAR of FFF machines in a large-scale AM system can provide a new opportunity to maximize the overall performance of the AM system.

## Figures and Tables

**Table 1:** Varied and fixed process parameters for experiments.

Type	Process Parameter	Value
Varied	Layer thickness (mm)	0.1 (L1), 0.15 (L2), 0.2 (L3), 0.25 (L4), and 0.3 (L5)
	Printing speed (mm/min)	1000 (P1), 1100 (P2), 1200 (P3), 1300 (P4), 1400 (P5), and 1500 (P6)
Fixed	Bed temperature (°C)	120
	Nozzle temperature (°C)	510
	Nozzle diameter (mm)	0.4
	Perimeter shells (# of layers)	3
	top layers/bottom layers (# of layers)	None
	Extrusion percentage for the first layer (%)	96
	Infill pattern (pattern)	Rectilinear
	Infill density (%)	100

**Table 2:** Collected data from experiments.

Name	Definition	Unit
Build time (*t*)	The total time taken during the material extrusion stage	s
Deposited material volume (*v*)	The total volume of deposited material during the material extrusion stage	mm^3^
Energy consumption (*EC*)	The total energy consumed during the extrusion stage	W·h
Dimensional accuracy (*DA*)	The root mean square error of a final output between original and measured dimensions	mm

**Table 3:** Regression models for energy consumption.

Sample Type	Regression Model	*R*-Squared	$\mathrm{Test}\;\mathrm{for}\;\beta_{0(EC)}$ (*p*-Value)	$\mathrm{Test}\;\mathrm{for}\;\beta_{1(EC)}$ (*p*-Value)
Sample A	$\mathrm{EC}=11.43+55.90\cdot \mathrm{MA}R_{\mathrm{inv}}$	99.74%	*t* = 33.51 (*p* = 0.00 **)	*t* = 183.03 (*p* = 0.00 **)
Sample B	$\mathrm{EC}=13.49+108.36\cdot \mathrm{MA}R_{\mathrm{inv}}$	99.82%	*t* = 15.60 (*p* = 0.00 **)	*t* = 126.21 (*p* = 0.00 **)
Sample C	$\mathrm{EC}=15.25+109.47\cdot \mathrm{MA}R_{\mathrm{inv}}$	99.68%	*t* = 12.26 (*p* = 0.00 **)	*t* = 93.24 (*p* = 0.00 **)

** *p* < 0.05.

**Table 4:** Regression models for dimensional accuracy.

Sample Type	Regression Model	*R*-Squared	$\mathrm{Test}\;\mathrm{for}\;\beta_{0(DA)}$ (*p*-Value)	$\mathrm{Test}\;\mathrm{for}\;\beta_{1(DA)}$ (*p*-Value)
Sample A	DA=0.31+0.27·MAR	49.94%	*t* = 9.55 (*p* = 0.00 **)	*t* = 9.37 (*p* = 0.00 **)
Sample B	DA=0.23+0.29·MAR	51.00%	*t* = 3.36 (*p* = 0.002 **)	*t* = 5.40 (*p* = 0.00 **)
Sample C	DA=0.13+0.43·MAR	65.02%	*t* = 1.73 (*p* = 0.094)	*t* = 7.21 (*p* = 0.00 **)

** *p* < 0.05.

**Table 5:** Derived optimal MAR for overall AM performance.

Sample Type	Normalized EC Model	Normalized DA Model	AM Performance Model	Optimal MAR
Sample A	$EC_{\mathrm{norm}}=1.37–0.67/\mathrm{MAR}$ - Test for $\beta_{0}$(*p*-value): 0.00 - Test for $\beta_{1}$(*p*-value): 0.00 - *R*-squared: 99.74%	$DA_{\mathrm{norm}}=0.97–0.34\cdot \mathrm{MAR}$ - Test for $\beta_{0}$(*p*-value): 0.00 - Test for $\beta_{1}$(*p*-value): 0.00 - *R*-squared: 49.94%	*P* = 2.34–0.67/*MAR*–0.34·*MAR*	1.40
Sample B	$EC_{\mathrm{norm}}=1.36–0.74/\mathrm{MAR}$ - Test for $\beta_{0}$(*p*-value): 0.00 - Test for $\beta_{1}$(*p*-value): 0.00 - *R*-squared: 99.82%	$DA_{\mathrm{norm}}=1.12–0.44\cdot \mathrm{MAR}$ - Test for $\beta_{0}$(*p*-value): 0.00 - Test for $\beta_{1}$(*p*-value): 0.00 - *R*-squared: 51.00%	*P* = 2.48–0.74/*MAR*–0.44·*MAR*	1.30
Sample C	$EC_{\mathrm{norm}}=1.35–0.68/\mathrm{MAR}$ - Test for $\beta_{0}$(*p*-value): 0.00 - Test for $\beta_{1}$(*p*-value): 0.00 - *R*-squared: 99.68%	$DA_{\mathrm{norm}}=1.26–0.67\cdot \mathrm{MAR}$ - Test for $\beta_{0}$(*p*-value): 0.00 - Test for $\beta_{1}$(*p*-value): 0.00 - *R*-squared: 65.02%	*P* = 2.61–0.68/*MAR*–0.67*·MAR*	1.01

**Table 6:** Suggested process parameter settings.

Suggested Process Parameters	MAR	Measured EC	Measured DA
Sample A	1.40 (optimal)	51.4 (estimated)	0.69 (estimated)
L4 and P4	1.38	52.2	0.66
L4 and P5	1.42	50.9	0.64
L5 and P1	1.37	53.5	0.74
Sample B	1.30 (optimal)	96.8 (estimated)	0.61 (estimated)
L3 and P4	1.27	98.2	0.63
L3 and P5	1.34	93.8	0.67
L4 and P1	1.28	99.2	0.72
Sample C	1.01 (optimal)	123.6 (estimated)	0.56 (estimated)
L2 and P5	0.99	126.3	0.35
L2 and P6	1.04	122.1	0.53
L3 and P1	0.99	129.3	0.65

## Data Availability

The data presented in this study are available on request from the corresponding author. The data is not publicly available due to privacy or ethical restrictions.
